# COVID-19 Vaccination Prioritization Strategies in Malaysia: A Retrospective Analysis of Early Evidence

**DOI:** 10.3390/vaccines11010048

**Published:** 2022-12-26

**Authors:** Nor Elyzatul Akma Hamdan, Mathumalar Loganathan Fahrni, Antonio Ivan Lazzarino

**Affiliations:** 1 Faculty of Pharmacy, MARA University of Technology (UiTM), Selangor Branch, Puncak Alam Campus, Puncak Alam 42300, Malaysia; 2 Department of Epidemiology and Biostatistics, School of Public Health, Imperial College London, London W2 1NY, UK

**Keywords:** COVID-19 vaccines, high-risk, equitable access, risk stratification, SARS-CoV-2, health service accessibility, global health, health equity, immunization programs, inoculation, low- and middle-income countries

## Abstract

The coronavirus disease 2019 (COVID-19) that can cause extreme acute respiratory syndrome has posed a catastrophic threat to public health. The vaccines had indeed restored optimism and, after more than two years of battling the pandemic, there is renewed hope for the transition to endemicity. At the start of vaccination efforts, when supply shortages of vaccines were inevitable, every nation determined the high-risk population groups to be given priority for the COVID-19 vaccines. In this paper, the characteristics of the initial COVID-19 vaccine recipients in Malaysia are described. In line with the policies of many other countries, Malaysia firstly inoculated frontline healthcare workers, and subsequently the list of front liners grew to include defense and security personnel and those involved in the provision of essential services. People with disabilities or those with special needs and several underlying medical conditions that increased their risk of developing severe COVID-related illnesses were included in the priority categories. These included patients with severe lung disease, chronic heart disease, chronic kidney disease, chronic liver disease, neurological disease, diabetes mellitus and obesity in adults, splenic dysfunction, and severe mental illness. With little information and under circumstances of great uncertainty, the Health Ministry of a middle-income country had developed a vaccination priority-list based on the disease’s epidemiology and clinical data, vaccine type, operational considerations, and risk evaluation. Early evidence was presented and suggested that the full vaccination with any of the three predominant vaccines (AZD1222, BNT162b2, and CoronaVac) in the country had been highly effective in preventing COVID-19 infections, COVID-19-related ICU admissions, and death. As many SARS-CoV-2 variants of concern (VoC), such as the Omicron BA.2/4/5, are emerging, future vaccination strategies may necessitate the need to change the immunogen of the vaccine, as well as considerations for when to give high-risk groups booster injections. These considerations are valuable for future planning by policymakers and healthcare providers to make vaccination policy and decisions, especially for the inclusion of the COVID-19 vaccines into national immunization programs.

## 1. Background

The coronavirus disease 2019 (COVID-19) that can cause extreme acute respiratory syndrome has posed a catastrophic threat to public health [1]. The disease is caused by severe acute respiratory syndrome coronavirus 2 (SARS-CoV-2), a strain of the coronavirus [2]. Although the most likely ecological reservoirs for SARS-CoV-2 are bats, it is believed that the virus overcame the species barrier to humans through another intermediate animal host. This intermediate host, which remains unidentified could be a live, wild or domesticated animal species, or animal products. SARS-CoV-2 is a Betacoronavirus that causes fever, unproductive cough, myalgia, and fatigue. It belongs to the Coronaviridae family of viruses [2]. After about a year of battling COVID-19, the COVID-19 vaccines were developed, giving the world a new lease on life and renewed hope. Malaysia intended to vaccinate at least 80% of the population in less than a year to achieve herd immunity [3]. Immunization is necessary to protect the general public, especially those at high risks for developing severe COVID-19, such as the elderly and those with medical conditions. Patient safety and vaccine efficacy were prime concerns in addition to achieving broad vaccination coverage in a short period. After the first vaccine products were licensed and at a time when supply shortages were unavoidable, countries all over the world had the difficult task of deciding which population groups ought to be given a priority for the COVID-19 vaccination. Hence, we aimed at reviewing the essential characteristics of COVID-19 vaccine recipients in Malaysia, which will serve as relevant information in preparation for a future pandemic and thus assist healthcare providers in making vaccination decisions, particularly for high-risk groups. The review comprehensively summarized the characteristics of COVID-19 vaccine recipients in Malaysia, vaccine distribution strategies, the characteristics of targeted high-risk groups, as well as approaches for procuring and using the COVID-19 vaccines. The definition of high-risk groups is very instructive for other countries to make similar decisions.

## 2. Methods

Data were collected following a comprehensive search strategy using online databases. The data were extracted from the following databases: Science Direct, Scopus, and EBSCO: MEDLINE. The search was carried out using relevant keywords that were directly linked with the topic of interest with various synonyms. The search was restricted to the English language and focused on the period from March 2020 to March 2021. The two Boolean operators OR and AND were used, for example, “coronavirus disease 2019 * COVID-19 * AND (OR Vaccination* Immunization)” AND “(Malaysia*)” AND “(Priori* Strategies OR Hierarchy)”. Manual searching of bibliographies was carried out to find other relevant studies. Full texts and metadata were imported to the Zotero program to assist with data collection and to remove duplicates.

## 3. Results and Discussions

### 3.1. COVID-19 Vaccination Policy

#### Vaccine Distribution Strategy and Target Groups

The COVID-19 vaccination is voluntary and was provided free of charge to all Malaysians, including citizens and non-citizens. The government aimed to inoculate at least 80% of Malaysia’s population with the vaccines by February 2022 to lessen infections, hospital admissions, and fatalities. [4]. A special committee, known as the Special Committee for Ensuring Access to COVID-19 Vaccine Supply, *Jawatankuasa Khas Jaminan Akses Vaksin* COVID-19 (JKVAV), was responsible for the COVID-19 vaccine supply. Alongside the JKJAV, the National Pharmaceutical Regulatory Agency (NPRA) played a vital role in ensuring vaccines’ efficacy, quality, and safety. Following waves of rising infections, as of 10 February 2022, 98% of the country’s adult population eligible for vaccination had completed the required doses and were successfully inoculated, while 53.9% had received their booster shots. The plan to vaccinate adolescents and toddlers as young as 5 and other special interest population groups was also rolled out in phases across the country depending on vaccine manufacturers’ supplies and deliverables [5].

According to the National COVID-19 Immunization Programme [4], the first strategic approach was to vaccinate frontliners, particularly those in the healthcare sector, in order to protect them from debilitating infection. This strategy was also aimed at ensuring that the healthcare system continued to function optimally. The second aim was to minimize disease burden in high-risk groups against COVID-19-associated diseases. This was done to reduce the strain on Malaysia’s public health system. The third step to contain the pandemic was for vaccination to be conducted in phases two and three in high-risk areas, based on risk assessment. COVID-19 vaccination yielded positive results, such as decreased new severe COVID-19 cases, fewer admissions to intensive care units, and decreased rates of mortality in the country. Upon vaccinating 600,000 of its citizens, the vaccine’s effectiveness resulted in a 94 percent reduction in COVID-19 infections in the vaccinated group [6]. The Ministry of Health had developed a vaccination priority list based on the disease’s epidemiology and clinical data, vaccine type, operational considerations, and risk evaluation [3].

The first phase of vaccination, which targeted 500,000 people, was initiated in February 2021 and lasted until April 2021. This phase focused on Group 1 priority group, which consisted of those who worked as frontliners in the public and private healthcare sectors, while priority Group 2 frontliners included personnel from essential services, defense, and security. As National Academy Medicines and others have suggested, prioritizing in-person healthcare workers and staff can prevent direct and indirect harm to workers due to the spread of SARS-CoV-2 in healthcare facilities. It also benefited disadvantaged groups indirectly because reducing disease spread made it easier to provide treatment and essential services [7]. The JKJAV committee was responsible for updating the list of those who provided essential services from time to time.

The second phase, which targeted 9.4 million people, was initiated in April 2021 and lasted until August 2021. Group 1 priority group was maintained and consisted of healthcare workers, those on the essential services list, as well as defense and security personnel. Group 2 priority group consisted of senior citizens (those aged 60 and over), people with chronic diseases (such as heart disease, obesity, diabetes, and high blood pressure), and people with disabilities or special needs. The data on people with disabilities were reviewed regularly by the JKJAV committee.

Prioritizing people engaged in high-risk yet essential activities such as in-person education, childcare, and food chain supply reduced the spread of the disease and prevented direct harm. Additionally, in-person workers were more likely disadvantaged socioeconomically than those who could work remotely. Prioritization among these workers also indirectly benefited them: for example, it helped those in education to have immunity against COVID-19 so they could work on reopening schools.

The World Health Organization (WHO) and the National Academy of Medicine (NAM) recommended the prioritization of patients with medical conditions and who already had poor prognoses. The consequences of COVID-19 infections for them would likely have been life-threatening [8]. In addition, disadvantaged groups with medical vulnerabilities were likely linked to socioeconomic disadvantage, and hence it was crucial to protect them from harm [9]. Due to limited vaccine supplies, those people were prioritized, with an eye kept on new information about how certain conditions affected risks of COVID-19 infection and vaccine efficacy.

The third phase of vaccination, which targeted more than 13.7 million people, lasted from 20 May 2021 until 20 February 2022. Priority groups at this stage were the adult population defined as anyone over 18 years old (both citizens and non-citizens). Those in the red zones were given priority, followed by those in the yellow zones and green zones [3]. Areas were classified as “red zones” when 41 or more COVID-19 cases were registered within the area for a 14-day period. “Yellow zones” were areas where one to 40 COVID-19 cases were registered, and “green zones” were areas where there were no reported cases of COVID-19. Since the implementation of the conditional movement control order (CMCO) and appropriate zoning of districts with high number of cases, the Health Ministry had reportedly seen an improvement in the R-naught number. R-naught (R0) is a value that can be calculated for communicable diseases that represents, on average, the number of individuals to whom a single sick individual is likely to spread the disease. R0 is a computation of the average “spreadability” of an infectious disease [10].

In September 2021, the COVID-19 Immunisation Task Force-Adolescent (CITF-A) was formed under the Ministry of Health to target full vaccination for 80% of Malaysians aged 12 to 17 years before the reopening of schools. The committee was tasked with overseeing the National COVID-19 Immunisation Programme for Adolescents (PICK Adolescents) which were conducted via a scheduled walk-in process. The program included students in the private education sectors, adolescents in protection and rehabilitation programs, as well as the refugee communities, homeless, and non-citizens in Malaysia.

### 3.2. Characteristics of COVID-19 Vaccine Recipient of Priority

A clinical guideline on COVID-19 vaccination was prepared as a reference for health personnel in evaluating a person’s suitability to receive the vaccine. The Ministry of Health periodically updated the list of preventive measures and high priority groups (Table 1) [10].

The following are the characteristics of priority groups [11] according to the Clinical Guideline on COVID-19 Vaccination in Malaysia:

#### 3.2.1. Immunocompromised as a Result of Disease or Treatment

Because mRNA vaccine is not a live vaccine, it is not contraindicated for immunocompromised patients [12]. Immunocompromised patients are particularly vulnerable to severe COVID-19-related illnesses [13]. Nevertheless, there is inadequate information on vaccine efficacy. To balance vaccine efficacy and timely protection against COVID-19 infections, the health care provider must determine the optimal timing of vaccination after consultation with patients [3].

The first group comprised patients who received a bone marrow or stem cell transplant, and any solid organ transplant. Before vaccinations, the recommended timing and preparations should be at least three months after the transplant [14]. The second group of immunocompromised patients are patients diagnosed with haematological malignancies. It is recommended to wait until absolute neutrophil count (ANC) recovery in those receiving intensive cytotoxic chemotherapy. However, vaccination is recommended as soon as the vaccine is available for long-term therapy or for those expected to have limited or no recovery from marrow failure [15]. It is best to discuss the mutual timing for vaccination and cancer therapy with the patient’s health care provider [3]. Additionally, immune system disorders caused by genetic mutations, (such as systemic lupus erythematosus, rheumatoid arthritis, and psoriasis) that necessitated long-term immunosuppressive therapy were a focus as well. These involved adults taking systemic steroids daily for more than a month, and individuals who were prescribed with immunosuppressive biological therapy [16].

#### 3.2.2. Human Immunodeficiency Virus Infection

Priority groups for vaccination included human immunodeficiency virus (HIV)-positive individuals on antiretroviral therapy and with additional underlying medical conditions that raised the risk of serious infection from COVID-19. There seem to be limited data on vaccine efficacy in HIV-positive individuals whose conditions were not well-controlled by medication [17]. According to a report conducted in 2021, HIV doubled the mortality risk from COVID-19, and a history of tuberculosis had a similar effect [18]. Cohort studies in the United States, the United Kingdom, and South Africa have recorded worsened patient outcomes for HIV and COVID-19 coinfections, including increased mortality rates [18,19,20]. People living with HIV have a much greater risk of contracting COVID-19 and Hepatitis C co-infections due to their impaired immune systems. Super added COVID-19 infection may prove to be fatal in these patients. The most prevalent comorbidity among HIV-positive patients co-infected with COVID-19 was cardiovascular illness (27.2%), with hypertension accounting for 23.9% of cases. This was followed by diabetes mellitus (12.2%), chronic lung disease and asthma (4.2% each), and lifestyle-related illnesses such as dyslipidemia and obesity (5.4%), smoking, drinking, and illegal drug use (2.9%, 0.7%, and 1.4%, respectively). Other notable comorbidities include chronic renal disease (6.4%) and liver disease (1.4%) [21].

Lower CD4+ T lymphocyte levels were linked to a higher risk of intensive care unit admission, mechanical ventilation, or death in a large prospective cohort study of 286 HIV and COVID-19 co-infected patients in the United States. This also applied to patients who had achieved HIV virologic suppression where an increased risk was still found [22]. Additionally, a low CD4+ T count was linked to poor outcomes in another study of 175 patients with both HIV and COVID-19 [23]. According to a survey performed in New York, people with HIV who tested positive with COVID-19 had increased admission rates to the hospital and death than people with COVID-19 who did not have HIV [24].

#### 3.2.3. Pregnancy

Although available data now show no increased risk to pregnancy, there then seemed to be limited evidence to support the scheduled use of COVID-19 vaccines during pregnancy. As a result, vaccinations during pregnancy were done after counselling and as part of a shared decision-making process [25].

Vaccines were recommended for those at high risk of SARS-CoV-2 infection, including healthcare professionals or women with underlying conditions that increase the likelihood of them contracting a severe infection from COVID-19. In addition to the health issues outlined, pregnant women with the following characteristics [11] were considered for vaccination: age greater than 40, hemoglobin levels less than 7 g/dL, and smoking.

Even though clinical trials during the development of COVID-19 vaccines did not include pregnant women, current scientific knowledge implies that COVID-19 vaccinations among pregnant and breastfeeding women are likely to be safe [3]. There are no known risks associated with administering inactivated virus or bacterial vaccines or toxoids during pregnancy or while breastfeeding. It was unnecessary to routinely enquire about one’s last menstrual period or perform pregnancy testing [3]. Pregnant women who chose vaccination received their first dose between 14 and 33 weeks of a pregnancy. In other cases, vaccinations for diseases such as influenza, tetanus, diphtheria, and pertussis (TDaP) and for monkeypox can be given 14 days apart [26]. If pregnant mothers conceive after the first dose, they may choose to postpone the second dose until after 14 weeks [27]. Moreover, pregnant women who received vaccines such as TDaP showed no evidence of foetal harm [28]. Pfizer-BioNTech and Moderna are both mRNA-based vaccines that produce “spike proteins” that mimic the surface protein of SARS-CoV-2 to elicit an immune response. Because these vaccines do not contain live viruses of SARS-CoV-2, they were not known to be infectious to the pregnant mother or foetus [29]. The developmental and reproductive toxicity (DART) study in pregnant and lactating females was completed to assess the potential fertility and pre and postnatal development effects [30]. The FDA reviewed this study and stated that mRNA-1273 given before mating as well as during reproductive cycles at a dose of 100 μg had no adverse effects on reproductive organs, fetal/embryonal development, or postpartum development, except for skeletal variations, which were common and usually resolved postpartum without treatment. Pregnancy-related animal data for the Pfizer-BioNTech vaccine appear to point in the same direction.

While the number of women who received the COVID-19 vaccine inadvertently while being pregnant is small, the results are encouraging. There were 13 pregnancies reported, with 6 in the mRNA-1273 arm and 7 in the placebo arm. Ten of the thirteen pregnancies went smoothly without problems. One patient in the placebo group had a spontaneous miscarriage at seven weeks, another had an elective termination at six weeks, and another patient was loss to follow up. In contrast, the study reported no adverse effects in the mRNA-1273 group. The Center for Disease Control and Prevention (CDC) has created a smartphone app called “v-safe” to collect side effect reports from immunized people. At the time of writing, approximately 15,000 pregnant women had enrolled in the registry, which will likely provide more evidence of its safety [31].

#### 3.2.4. Lactating Women

Many lactating women, such as front-line healthcare workers, were in the high-risk groups for immunization. Both the WHO Interim Guidance on the use of mRNA-1273 (Moderna) and the Academy of Breastfeeding Medicine do not suggest that lactating mothers who were vaccinated against COVID-19 stop breastfeeding [3]. There is currently little data for nursing mothers, as pregnant women were excluded from COVID-19 vaccine trials. Nevertheless, there is very little biological evidence that the vaccine will be harmful, and antibodies to SARS-CoV-2 in breast milk may in fact safeguard the breastfeeding child [32].

The vaccine comprises lipid nanoparticles containing mRNA for the SARS-CoV-2 spike protein, which triggers an immune response and protects the individual from COVID-19 infection. It is unlikely that the vaccine lipid would enter the bloodstream and reach breast tissue during lactation. If it does, neither the intact nanoparticle nor the mRNA is likely to transfer into milk. In the unlikely scenario that mRNA is available in milk, it is expected to be digested by the infant and seems to have no biological effects [33]. While the child faces little risk, there is some scientific evidence that antibodies and T-cells displaced by the vaccine may be transferred passively into milk [34]. IgA antibodies are detectable in milk 5 to 7 days after vaccination against other viruses. Antibodies transmitted into milk may help shield the newborn baby from contracting the SARS-CoV-2 infection [32].

#### 3.2.5. Thrombocytopenia, Blood Disorders, and Coagulation Disorders

Patients with known thrombocytopenia (platelet counts less than 50,000) can receive the vaccination without additional hemostatic support. However, in that case, they should postpone the vaccination until their platelet counts recover, if possible [35].Vaccination for those with chronically low platelet counts should be done in consultation with their primary hematologists. Patients who have stable anticoagulation and an INR of less than four on their most recent scheduled visit can receive IM vaccination without discontinuing their medication [36]. Patients taking warfarin and antiplatelet therapy should be managed personally by their primary care physicians. Patients should take warfarin after the vaccine injection [37]. Patients taking dual oral-anticoagulation (DOAC), low-molecular weight heparin (LMWH), or fondaparinux can postpone their daily dose until after the intramuscular injection on the day of vaccination. Still, they do not have to skip any dose [38]. In rare bleeding disorders, including platelet function disorder, they should be vaccinated after consulting with their primary hematologists [3].

#### 3.2.6. The Underlying Medical Conditions That Raise the Risk of COVID-Related Severe Illness

##### Individuals Suffering from a Severe Lung Condition

Patients suffering from a severe lung disease such as asthma who require repeated and prolonged use of systemic steroids or had proceeding exacerbations which necessitated emergency room visits, as well as those with chronic obstructive pulmonary disease (COPD), along with chronic bronchitis and emphysema, bronchiectasis, cystic fibrosis, interstitial lung fibrosis, pneumoconiosis, and benign prostate disease belong in this group [3]. Asthma patients had higher COVID-19-related medical costs and a higher proportion of them experienced respiratory failure, which necessitated artificial ventilation or extracorporeal membrane oxygenation (ECMO) compared to non-asthmatic patients. The group’s median age and number of comorbidities were higher; the duration of stay and frequency of ICU admission were also higher. These potentially contributed to higher medical costs [39]. In the previous year, patients who had an acute asthma exacerbation had a more than twofold higher chance of death than those who never had an exacerbation. The existing state of asthma affected exacerbations [40]. Furthermore, acute exacerbations resulted in increased emergency department visits or hospitalizations, raising the risks for COVID-19 infections. As a result, asthma patients who recently had an exacerbation should be considered at high-risk with a poor prognosis for COVID-19 [41].

##### Cardiovascular and Chronic Heart Diseases

Individuals with congenital heart disease, hypertension with cardiac abnormalities, chronic heart failure, congenital heart disease, arrhythmia, peripheral vascular disease, or a history of pulmonary embolism are all at risk [3]. In patients infected with the COVID-19 predecessors such as severe acute respiratory syndrome coronavirus (SARS-CoV) and Middle East respiratory syndrome-related coronavirus (MERS-CoV), cardiovascular diseases (CVD) were severe comorbidities. Cardiovascular diseases were prevalent in 8% of the patients with SARS, and the prevalence of comorbid conditions raised the risk for mortality 12-fold [42]. Hypertension was found in 50% of MERS cases, and cardiovascular diseases were found in 30% of patients [43]. In a study of 191 patients from Wuhan, China, 48 percent (67 percent of non-survivors) had some comorbidity, 30 percent (48 percent of non-survivors) had hypertension, and 8% had CVD (13 percent of non-survivors) [44]. In a cohort of 138 COVID-19 hospitalized patients, comorbidities were equally prevalent. 72% were admitted to intensive care unit (ICU), and had cardiovascular comorbidities: 31% had hypertension (58% of the patients had ICU care), and 15% had CVD (25% receiving ICU care) [45]. An analysis of 1099-patient outpatients and inpatients showed that 24% had some comorbidity (58 percent among those who required intubation or who ended up with death as an outcome), 15% had hypertension (36% among those who required intubation or who ended up with death as an outcome), and 2.5 percent had coronary heart disease (9 percent among those with intubation or who ended up with death as an outcome) [46].

##### Chronic Kidney Disease

Chronic kidney disease in stages 3, 4, or 5, chronic renal failure, nephrotic syndrome, and kidney transplant are all potential medical conditions that were prioritized for vaccination [3]. One potential reason for the high prevalence of kidney impairment upon hospitalization is that those COVID-19 patients already had a history of chronic kidney disease. These patients had proinflammatory conditions with structural deficiencies in the innate and adaptive immune cells and have an increased chance of acquiring upper respiratory tract infection and pneumonia [46,47,48]. One study observed that patients with increased baseline serum creatinine were more likely to end up in the ICU and required mechanical ventilation. Kidney failure posed a higher risk for deterioration. Previously, kidney damage was linked to increased mortality in patients with influenza A subtype H1N1 and SARS [49]. In another study, kidney failure was associated with a greater incidence of in-hospital mortality after adjusting for possible confounders [50].

##### Long-Term Liver Disease

Cirrhosis, biliary atresia, and chronic hepatitis are all symptoms of a failing liver. Because of the overwhelming inflammatory responses, patients with those symptoms can experience an acute-to-chronic liver failure [51]. Patients with liver cirrhosis are at a greater risk of secondary bacterial infection and a more severe course of influenza, which may lead to organ failure, secondary infections, and death [52]. Diabetes, hypertension, and obesity are associated with an extreme course of COVID-19 in patients with non-alcoholic fatty liver disease (NAFLD) or steatohepatitis [53]. One study looked at 14 patients with SARS-CoV-2 infection and pre-existing NAFLD, six of whom had severe disease and worse outcomes [54]. According to another study, patients with NAFLD had a higher chance of progressing to extreme COVID-19 and a prolonged viral shedding duration [55].

##### Neurological Disease

A chronic neurological disease included stroke and transient ischemic attack (TIA). Patients with cerebral palsy, severe-to-profound learning disabilities, mental retardation, multiple sclerosis, seizures, Alzheimer’s disease, Parkinson’s, muscular dystrophy, and relevant conditions are at high risk. Patients with neurological disorders who require therapies such as immunosuppressant drugs for autoimmune neurological diseases, may experience exacerbated outcomes from COVID-19 [56]. Previous reports indicated that with the use of corticosteroids, viral shedding can be prolonged. Virologists consider viral particles being shed as infectious. During COVID-19, however, the definition of shedding had been broadened to include the shedding of viral genetic material (RNA). Although RNA constitutes fragments of the virus, these are not necessarily infectious fragments. Studies measuring the shedding of viral genetic material from the respiratory tract have reported that shedding typically lasts around 17 days [44,57]. Para-infectious neurological diseases such as Guillain–Barré syndrome, transverse myelitis, or acute disseminated encephalomyelitis, such as those seen in the 2015–2016 Zika virus outbreak, but on a much larger scale considering the number of people infected, could be a more significant concern for a direct viral invasion of the central nervous system (CNS). Patients with neurological problems may need to remain in intensive care for an extended period, putting more pressure on already overburdened facilities [56].

##### Type 1 or Type 2 Diabetes Mellitus and Obesity Adults with a BMI of More Than 30 kg/m^2^

Diabetes patients were considered more susceptible to a variety of acute and chronic infections than non-diabetic patients [58]. Diabetic patients were 3.1 times more likely than non-diabetic patients to have been admitted to the intensive care unit (ICU), needed artificial ventilation, or died during the pandemic [59]. Diabetes was found to triple the risk of hospitalization and quadruple the risk of ICU admission after influenza A (H1N1) [43]. Diabetes was also considered a risk factor for developing into extreme cases of the Middle East respiratory syndrome coronavirus (MERS-Cov) infections [60]. With the COVID-19 outbreak, a large percentage of diabetic patients were also found. It was the most common underlying comorbidity among nonsurvivors of critically ill conditions [61]. Diabetic patients had a higher mortality rate in the most extreme cases recorded by the Chinese Center for Disease Control and Prevention, which included 72,314 COVID-19 cases (7.3 percent in diabetes versus 2.3 percent overall) [62]. According to recent data from Italy, diabetes was present in more than two-thirds of those who died from COVID-19 [63]. Some clinical studies examined the biochemical characteristics of COVID-19 patients with diabetes and found that those with diabetes were more prone to developing severe or critical complications with higher mortality rates than those without diabetes and that both diabetes and hyperglycemia were independent predictors of COVID-19 fatality [64,65].

##### Splenia or Splenic Dysfunction

This applies to those with a splenectomy and those with conditions that can lead to splenic dysfunction, including thalassemia major and coeliac syndrome. Individuals with a damaged or defective spleen were more susceptible to bacterial sepsis caused by encapsulated pathogens [66]. Because of the elevated risks for secondary bacterial infections after contracting influenza, asplenic/hyposplenism patients were recommended to get an influenza vaccine. Patients who were usually eligible for regular vaccinations were now classified as “vulnerable,” meaning they were at moderate risks for COVID-19 complications, and as such they were advised to seek vaccination and maintain strict social distancing [67].

##### Severe Mental Illness

Individuals who had a mental illness or were diagnosed with bipolar or any psychiatric disorder causing severe dysfunctions were two or three times at a higher risk for contracting COVID-19 than the general population [68]. They were also more likely than the general population to have been obese or to have had other chronic disorders, including cardiovascular disease, type 2 diabetes, and diseases of the respiratory tract, all of which were risk factors for poor COVID-19 outcomes [69]. It is likely that the increased risk of poor COVID-19-related outcomes in people with psychiatric disorders, especially in those with serious mental illness, was due to other preexisting comorbidities linked to poor COVID-19 outcomes [70]. Severe mental disorder has been linked to immune dysfunction revealing a proinflammatory state and maladaptive T-cell activity [71,72]. Childhood adversity is linked to an increased risk of developing a particular mental illness, one factor linked to dysregulated immunological function [73]. Additionally, severe mental illness is also related to certain socioeconomic risk factors for infection [74]. In comparison to those with less severe mental illnesses and the general population, people with severe mental illness were more likely to have a socioeconomic disadvantage, employment without healthcare benefits, or jobs with precarious working conditions, and live in poverty, according to a study [75]. Most of them were confined in a facility where SARS-CoV-2 infectivity and transmission were high. People with serious mental illnesses were more often socially isolated, making it difficult for them to get the treatment and help they needed when they were sick [76].

### 3.3. Approaches for Procuring and Using the COVID-19 Vaccine

The need for a safe and effective vaccine was a national means to ensuring that the National COVID-19 Immunization Programme could be enforced to combat the pandemic. By leveraging its diplomatic relations and strategic international cooperation with other countries, vaccine manufacturers, and world health bodies and affiliates, the Malaysian government had taken a systematic approach in its vaccine acquisition efforts. The government, led by the Ministries of Health (MOH), Foreign Affairs (MFA), and Science, Technology, and Innovation (MOSTI), used science diplomacy efforts to secure access to a portfolio of COVID-19 vaccines that were being actively developed around the world. Whether multilateral or bilateral, strategic international cooperation was a concrete step by the country to join the national aspirations and participation in global solidarity efforts to combat COVID-19. It had allowed the government to expand its vaccine procurement options and reduce its reliance on a single supply source. Agreements with COVID-19 pharmaceutical companies were based on relevant elements of the vaccine, such as reliability, improvement, techniques, consistency, potential side effects, demographic target, protection, efficacy, dosing, registration, and availability, as well as the commercial aspects, including costing, reimbursements, delivery schedule, payment terms, and logistic support. In addition, the negotiation process involved mutual trust to secure the nation’s vaccine supply. This encompassed the future development of vaccine bottling (fill and finish), research and development (R&D), technology transfer, and vaccine manufacturing. As higher incidences of zoonotic spillover in an era of rapid deforestation of tropical areas and unprecedented wildlife trafficking occur, vaccines that are safe and effective are crucial countermeasures [77,78].

#### 3.3.1. Malaysia’s Participation in the COVID-19 Vaccines Global Access Facility (COVAX)

The Global Alliance for Vaccines and Immunization (GAVI), the Coalition for Epidemic Preparedness Innovation (CEPI), and the World Health Organization (WHO) launched the COVID-19 Vaccine Global Access (COVAX) facility as a global initiative aimed to provide equitable access to diagnostics, treatment, and vaccines throughout the world. Malaysia officially joined the COVAX Facility on 13 November 2020. The COVAX facility is a multi-pronged strategic approach allowing countries to broaden their viable alternatives and minimize the chances of relying solely on bilateral efforts to obtain COVID-19 vaccines [5]. Engagement within that institution ensured that the vaccines were available to Malaysians. Malaysia’s involvement also demonstrated a commitment in global solidarity to guarantee equitable access to COVID-19 vaccines and the relevant resources.

#### 3.3.2. Malaysian Chinese Bilateral Agreement

On 18 November 2020, the governments of Malaysia and the People’s Republic of China signed an agreement to collaborate on the safe and effective development of vaccines. This negotiation laid the foundation for Malaysia to gain priority access to the People’s Republic of China’s COVID-19 vaccines. It also enabled the sharing of knowledge and collaboration in science and technology between the two countries.

The United States has pledged support for the global vaccine procurement through the COVID-19 Vaccines Global Access (COVAX) initiative, which provided vaccines to low- and middle-income countries, but this funding was insufficient on its own [79]. By the end of 2021, COVAX hoped to have vaccinated at least 20% of the population of participating countries [79]. Even though this will be a significant accomplishment, it fell well short of the target of achieving global herd immunity in time [79]. Although pharmaceutical firms tended to regulate manufacturing regulations, suitable through voluntary licensing arrangements, pressure was mounting on the World Trade Organization to negotiate a Trade-Related Aspects of Intellectual Property Rights (TRIPS) waiver for COVID-19 vaccines [79]. This plan, proposed by India and South Africa, was recognized by more than 90 countries, and briefly lifted pharmaceutical patent rights, and lowered vaccine manufacturing costs worldwide [80]. A temporary waiver of intellectual property rights for the COVID-19 vaccine paved the way for a complete overhaul of the pharmaceutical patent system [79]. Many thought that it was timely that these inventions, such as medicines, vaccines, and medical devices, were removed from individual purviews patent rights and replaced with global property rights [81].

### 3.4. Impact of the Prioritization Strategy on Initial Mortality and ICU Admission

When Malaysia rolled out an initial portfolio of predominantly three COVID-19 vaccines (AZD1222, BNT162b2, and CoronaVac) beginning 24 February 2021, researchers evaluated vaccine effectiveness up until 15 September 2021 using two methods: (1) the screening method for COVID-19 infections, and (2) a retrospective cohort of confirmed COVID-19 cases for COVID-19 related ICU admission and death. Partial vaccination was estimated to have been 48.8% effective (95% CI: 46.8, 50.7) against COVID-19 infections while full vaccination was estimated to have been 87.8% effective (95% CI: 85.8, 89.7) against COVID-19 infections. Among the cohort of confirmed COVID-19 cases, partial vaccination with any of the three vaccines was estimated to have been 31.3% effective (95% CI: 28.5, 34.1) in preventing an ICU admission, and 45.1% effective (95% CI: 42.6, 47.5) in preventing death. Full vaccination with any of the three vaccines was estimated to have been 79.1% effective (95% CI: 77.7, 80.4) in preventing ICU admission and 86.7% effective (95% CI: 85.7, 87.6) in preventing deaths. Their findings suggested that full vaccination with any of the three predominant vaccines (AZD1222, BNT162b2, and CoronaVac) in Malaysia had been highly effective in preventing COVID-19 infections, COVID-19-related ICU admissions, and death [82].

## 4. Conclusions

In retrospect, at the onset of the pandemic, the COVID-19 vaccination program in Malaysia was based on priority groups. The review provided some validation that the government agencies charged with oversight of the plan for vaccine administration identified those individuals at greatest risk for exposure to the virus and/or who were most critical to front-line defense and security. There was an apparent communication between the Malaysian government through its designated agencies with the WHO and other countries to ensure their participation in the global efforts to contain the infections. Early evidence was presented to suggest that the full vaccination with any of the three predominant vaccines (AZD1222, BNT162b2, and CoronaVac) in Malaysia had been highly effective in preventing COVID-19 infections, COVID-19-related ICU admissions, and death.

The guidelines for the priority group were indeed created with little information and under circumstances of great uncertainty. The novel nature of the SARS-CoV-2 pathogen and the evolving epidemic, economic, and social circumstances presented challenges in determining priority groups for vaccine use at that time. Apart from unknown clinical and epidemiological factors, this recommendation and guidance make several plausible assumptions about vaccine characteristics. Furthermore, complex models of various priority circumstances gradually evolved, and evidence-based guidance is continuously shifting. For most of these purposes, the development plan may be revised and amended in light of new evidence. As many SARS-CoV-2 variants of concern have emerged, such as the Omicron BA.2/4/5, future vaccination strategies may necessitate the need to change the immunogen of the vaccine, as well as considerations for when to give high-risk groups booster injections. This information is valuable for future planning by policymakers and healthcare providers to make vaccination policy and decisions, especially for the inclusion of COVID-19 vaccination into national immunization strategies.

## Figures and Tables

**Table 1:** Vaccine priority groups and underlying medical conditions that increase the risk of severe illness from COVID-19 (adapted from Green Book, Public Health England, Chapter 14a, Covid-19).

Priority Groups ^a^		
1	Immuno-compromised due to disease or treatment	Bone marrow or stem cell transplant recipients
		Solid organ transplant recipients
		Haematological malignancies
		People with cancers undergoing active chemotherapy, immunotherapy, radiotherapy or other targeted therapy that result in immunosuppression
		Genetic disorders affecting the immune system
		Autoimmune diseases like SLE, RA and psoriasis who require long term immunosuppressive treatment
		Those who are receiving systemic steroids for >1 month at a daily dose equivalent to prednisolone 20 mg or more (for adults)
		Individuals who are receiving immunosuppressive or immunomodulating biological therapy such as anti-TNF, rituximab
2	HIV infection	Those with CD4+ T counts ≤350cells/mm^2^ or with additional underlying conditions that increase the risk of severe illness from COVID-19 are to be considered as priority groups for vaccination
3	Asplenia or dysfunction of the spleen	Those who have undergone splenectomy and those with conditions that may lead to splenic dysfunction, such as thalassemia major and coeliac syndrome
4	Chronic heart disease and vascular disease	Congenital heart disease, hypertension with cardiac complications, chronic heart failure, ischaemic heart disease, individuals with atrial fibrillation, peripheral vascular disease or a history of venous thromboembolism
5	Chronic kidney disease	Chronic kidney disease at stage 3, 4 or 5, chronic kidney failure, nephrotic syndrome, kidney transplantation
6	Chronic liver disease	Cirrhosis, biliary atresia
7	Chronic neurological disease	Stroke, TIA Individuals with cerebral palsy, severe or profound learning disabilities, Down’s Syndrome, multiple sclerosis, epilepsy, dementia, Parkinson’s disease, motor neuron disease and related or similar conditions; or hereditary and degenerative disease of the nervous system or muscles; or severe neurological disability. Conditions in which respiratory function may be compromised due to neurological disease
8	Chronic respiratory disease	Individuals with a severe lung condition, including those with asthma that requires continuous or repeated use of systemic steroids or with previous exacerbations requiring hospital admission, and COPD, including chronic bronchitis and emphysema; bronchiectasis, cystic fibrosis, interstitial lung fibrosis, pneumoconiosis and BPD
9	Diabetes mellitus	Type 1 or 2 DM
10	Obesity	Adults with a BMI ≥ 30 kg/m^2^
11	Severe mental illness	Individuals with schizophrenia or bipolar disorder, or any mental illness that causes severe functional impairment

^a^ Conditions listed here are in no order of priority.

## Data Availability

All relevant data are available in the article.
